# Idiosyncrasy

**Published:** 1902-06-28

**Authors:** 


### Idiosyncrasy.

The factor of idiosyncrasy in medical diagnosis and treatment appears to be in danger of being neglected altogether, but is, nevertheless, a very real and powerful element. In some medical schools the subject is scarcely alluded to at all, and in clinical hospital work the tendency is rather to regard the human organism as a machine which will inevitably react in a definite manner to definite methods of treatment. It is only in private practice that the physician fully realises that patients are distinct individuals who are affected in different manners by similar exciting causes, and frequently require varying methods of treatment even when suffering from the same disease. Now, although the element of imagination may modify the effects of treatment, it is an undoubted fact that whereas one person will be benefited by such drugs as quinine and salicylate of soda, another will be almost poisoned by them, even when given for symptoms apparently the same and in equal doses. Again, we find that a high temperature will affect people very differently, some being hilarious or sharp-tongued, while others are heavy, drowsy, and dull. One child may be playing with his toys with a temperature of 103°, while his brother is half comatose with a temperature of 101°. But, perhaps, the most extraordinary and interesting results are obtained when we systematically examine pulse rates. Some people will experience no inconvenience with a pulse rate of 120 or 140 to the minute, and others will be breathless and in evident distress when the rate rises to 90 or 100, although neither may have any organic disease to account for the symptom. Again, extraordinary varieties of symptoms are occasioned by that obscure condition which is called "lithæmia," "suppressed gout," or vaguely "liver." Some people are thereby rendered irritable and restless, others lethargic and sleepy, some lose their appetites, others eat voraciously; some complain of sharp pricks and pains in the back or joints, others experience a dull aching sensation. The condition itself may be due to injudicious or over eating, or it may be found in those overworked and badly nourished. It is more common in those who take but little exercise, but may be found in men who live a hard sporting life out of doors. Again, a light carbohydrate diet seems to suit some, but others can only exist on proteids, and the most widely different results are obtained by the use of the same drugs.

But it is in the functional nervous diseases that the greatest varieties of symptoms are found, and it is therefore in the treatment of this class of affections that the greatest experience is required. The thoughts and feelings of every neurotic are as different and distinct as are the features of the patient, although the same main factors may be constantly found.

We may, therefore, fearlessly state that the routine administration of valerian, assafœtida and other noxious drugs in these cases is practically useless, and that the only hope for successful treatment is by carefully studying each patient as an *individual* and not as a *case*. This is difficult, sometimes manifestly impossible in the out-patient room of an hospital, but can occasionally be attained in private practice. Some neurotics require rest and feeding, others occupation and dieting; to some electricity is the very essence of life, to others the sight of a battery will actually produce an hysterical fit; but to all the moral element in treatment is all important, and here the greatest tact is required. A clumsy practitioner who asked a young woman what she thought she was suffering from, and then proceeded that if it pleased her she might imagine she had any disease she wished, was doing his patient no good at all and only establishing for himself the reputation of being a "brute."

On the other hand, we all know that too much sympathy is also bad; but the happy mean has to be carefully considered for each case, and herein lies the difficulty in treatment. There is indeed no greater responsibility in the whole world than the use of the mind to cure the mind, and no greater satisfaction to the physician at a successful result, which is often rendered the more complete by the happy fact that the patient scarcely realises that he has been ill at all. Indeed, the greater the experience the more apparent it becomes that it is a dangerous practice to label certain symptoms by the name of a disease and then to prescribe from a list of drugs which have been successfully given for it at some time or other. The idea of the family doctor understanding the constitutions of his patients, and being therefore better able to treat them than a newcomer, has really a great element of truth in it, although it might be scoffed at by the modern house physician.

